# Virtual Reality and Internet of Things-Based Music Online Learning via the Graph Neural Network

**DOI:** 10.1155/2022/3316886

**Published:** 2022-10-11

**Authors:** Jian Lian, Yanan Zhou, Lina Han, Zhiguo Yu

**Affiliations:** ^1^School of Intelligence Engineering, Shandong Management University, Jinan 250357, China; ^2^Beijing College of Foreign Studies University, Beijing 100089, China; ^3^Department of Electrical and Automation, Shandong Labor Vocational and Technical College, Jinan 250022, China

## Abstract

Virtual reality and the Internet of Things have shown their capability in a variety of tasks. However, their availability in online learning remains an unresolved issue. To bridge this gap, we propose a virtual reality and Internet of Things-based pipeline for online music learning. The one graph network is used to generate an automated evaluation of learning performance which traditionally was given by the teachers. To be specific, a graph neural network-based algorithm is employed to identify the real-time status of each student within an online class. In the proposed algorithm, the characteristics of each student collected from the multisensors deployed on their bodies are taken as the input feature for each node in the presented graph neural network. With the adoption of convolutional layers and dense layers as well as the similarity between each pair of students, the proposed approach can predict the future circumstance of the entire class. To evaluate the performance of our work, comparison experiments between several state-of-the-art algorithms and the proposed algorithm were conducted. The result from the experiments demonstrated that the graph neural network-based algorithm achieved competitive performance (sensitivity 91.24%, specificity 93.58%, and accuracy 89.79%) over the state-of-the-art.

## 1. Introduction

Due to the global pandemic novel coronavirus [1], most of the pedagogical practices in all countries have to be carried out online, which significantly impacts or even hampers the expansion of teaching and learning activities [2]. Therefore, it is necessary to ensure the students concentrate on the content of courses during the process of learning. Meanwhile, the teachers also need to comprehend the dynamic changes of the entire online class. Both requirements have rendered the automatic identification of the status of the students become a thorny task.

In recent decades, virtual reality (VR) related techniques [3, 4] have been extensively exploited in a plethora of applications [5] ranging from industrial manufacturing [6], healthcare [7], entertainment [8, 9], and education [10, 11]. Meanwhile, different types of Internet of things (IoT) have also been deployed in practice [12], e.g., commercial and industrial scenarios [13, 14], medical assistance [15], and smart cities [16]. Both of them have shown their capacity and potential value in multidisciplinary tasks. However, online education especially music teaching remains a frontier domain that needs to be developed. Meanwhile, it requires an effective mechanism for measuring the students' academic performance since the adoption of the current evaluating manner might not be advisable.

Meanwhile, the deep learning-based models that emerged in the last years have been widely accepted as a powerful instrument in a plethora of domains and applications including but not limited to computer vision [17, 18], natural language processing [19], data mining [20], computer-aided diagnosis [21], recommendation system [22], and forecasting [23–26]. A variety of architectures were proposed in deep learning orientation, including the convolutional neural network (CNN) [27], recurrent neural network (RNN) [28], autoencoder [29], and generative adversarial network (GAN) [30]. It is worth noting that the deep learning-based algorithms focus on unveiling the inner patterns hidden in Euclidean samples and they usually neglect the non-Euclidean data with complicated associations and interdependency, e.g., social networks. Therefore, these deep models suffer from applying the common computational operators used in the CNN, RNN, autoencoder, and GAN to the graph domain.

On the other hand, to cope with the non-Euclidean data, numerous graph neural networks (GNNs) were put forward with different patterns and have shown their satisfactory performance in the recent period. As the work of Goodfellow et al. [30] is taken as an early work of GNN, the recently emerged GNNs can be roughly divided into four categories, including convolutional GNNs [31, 32], recurrent GNNs [33, 34], graph autoencoders (GAEs) [35, 36], and spatial-temporal GNNs [37, 38].

Although, plenty of GNN-based algorithms have been presented to cope with various machine learning tasks, e.g., handwritten signature recognition [31, 37], document discrimination [32, 35], ranking [33], program verification [34], and human activity detection [38]. In recent 5 years, for instance, Zhang et al. [39], proposed a deep graph clustering framework. First, a feature transformation module is built up of both the node and graph topology. Second, a graph embedding and a self-supervised learning mechanism are introduced to constrain graph embedding by using the graph similarity and self-learning loss. To deal with the molecular graph generation issue for drug discovery, Shi et al. [40] presented a flow-based autoregressive model, which combines both the autoregressive and flow-based approaches. Zheng et al. [41] proposed a graph multiattention network to predict traffic conditions for temporal phases ahead at differentiated positions within a traffic network graph. Aiming at addressing the fact-checking problem in the text, Zhong et al. [42] proposed a method for reasoning about the semantic level structure of evidence by using semantic role labeling. These algorithms have shown their performance in various scenarios. However, most of the GNN-based methods ignore feature information for each node in the graph.

Bearing the abovementioned analysis in mind, this work fills a gap in the literature by introducing VR integrated with IoT into musical pedagogy. To the best of our knowledge, this is also an early work for the employment of VR and IoT in online education. First of all, the proposed music teaching platform exploits the VR techniques to offer a uniquely immersive service for each student to experience an actual scenario without any hefty cost. For instance, the students can virtually perform in front of a large audience without a real stage. Second, it can collect the real-time status of the students by using IoT sensors. Accordingly, the students' physiological status including the heart rate, respiratory rate, temperature, and facial appearance can be captured. Third, the GNN model can then be introduced to represent the integral circumstance of the students to form the whole feature vector space of the online class. The personal information of all students captured from the IoT sensors is denoted as the nodes in a graph. The subjective judgment of the students' performance (positive or negative) made by the teachers is used as the ground truth in the training samples. Afterwards, a binary-class (positive and negative) graph neural network is trained over the manually labeled data samples. Accordingly, the subtle variation in this graph can be identified and predicted with high accuracy. The presented GNNs are then tested on the input samples and the practical condition of the students can be generated by updating the features embedded in the graph nodes. In general, the proposed network is supposed to alleviate the workload of the teacher in an online music class, while the network's output can unveil the status of each student revealed by the VR and IoT devices.

To evaluate the performance of the proposed framework, we conducted comparison experiments between state-of-the-art GNN models on the practical data samples collected from an online music class. Accordingly, the experimental results output from the proposed GNNs indicated that the GNN-based pipeline can come up with the practical engineering requirements.

In general, the contributions of this work consist of the following:

1. To our best knowledge, this is an early work for using VR and IoT in an online music learning platform
2. A semisupervised learning framework is proposed to recognize the students' status with GNNs. The situation of the entire class can be revealed prominently.
3. The experimental results demonstrate the effectiveness and efficiency of the proposed framework

## 2. Materials and Methods

### 2.1. Online Music Learning Framework and Dataset

**Figure 1:**
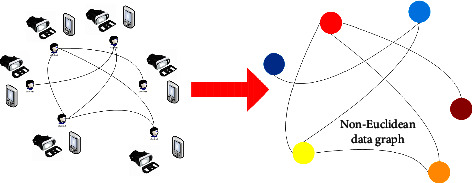
The pipeline from the online music learning platform with VR and IoT sensors to the non-Euclidean data structure.

An online music learning system (as shown in the left component of Figure 1) was built upon VR integrated with IoT sensors. This system consists of head-mounted VR display devices (Skyworth S801) and IoT sensors. The VR apparatus has 100° field of view and an optic lens. Before each class started, the students were instructed to wear both the VR apparatus and the wearable IoT sensors.

For each student, the data samples (heart rate, respiratory rate, temperature, and facial appearance) were collected and came into a feature vector at every 5 minutes. Then, three teachers labeled each sample as positive or negative separately by using a majority voting mechanism. In total, 6,104 recordings (3,020 positives and 3,084 negatives) from 16 students were collected in this study.

### 2.2. Graph Neural Network

#### 2.2.1. Problem definition

It is supposed that *n* subjects (i.e., the students as input samples) are available, denoted by S = [S_1_, S_2_, ..., S_n_]. Each student then can be denoted by one matrix Si ∈ R^m×ni^, where *m* represents the total number of IoT sensors mounted on each student, *n* is the length of the feature derived from the corresponding IoT sensor, and n_i_ ∈ {0, 1}. The weighted graph used in the proposed GNN is denoted as a tuple *G* = (V, E, W), where V is the set of *m* vertices, E represents the set of edges, and W ∈ R^m×ni^ is the adjacency matrix of the graph. Meanwhile, W_i,j_ represents the weight of the edge from v_i_ ∈ V to v_j_ ∈ V . In general, a global threshold is employed in the adjacency matrix to eliminate the irrelevant entries in the adjacent matrix or nodes in the graph. The space complexity of the proposed graph network is O(n^2^).

In general, the personal information of all students captured from the IoT sensors is denoted as the nodes in an undirected graph. Accordingly, the student's heart rate, respiratory rate, temperature, and facial appearance are integrated as a feature vector to represent the integral circumstance of the students. Meanwhile, the similarity between each pair of students is taken as the value of the edge between the two students denoted by two nodes. The edges with values lower than 0.4, which is set by conducting preliminary experiments, are eliminated from the graph.

In the proposed GNN model, the graph convolutional operator used can be considered as the spectral multiplication, which is equal to the convolution operator used in the temporal domain. Accordingly, the corresponding spectral filter can be realized by introducing the eigenfunctions of the normalized Laplacian into GNN.

(1) $$\begin{array}{c} L=I_{m}-D^{-1/2}WD^{-1/2}\text{,} \end{array}$$

where D denotes the degree of the matrix and I_m_ is an identity matrix. Furthermore, the Laplacian matrix can be implemented by using Chebyshev polynomials [43]:

(2) $$\begin{array}{c} T_{k}\left(L\right)=2LT_{k-1}\left(L\right)-T_{k-2}\left(L\right)\text{,} \end{array}$$

where T_0_(L) = 1 and T_1_(L) = L.

The K-ordered polynomial then yields unbiased K filters. Accordingly, the filtered outcome of the signal by K filters can be formulated as

(3) $$\begin{array}{c} o=g_{\theta }\left(L\right)*c=\underset{k}{\sum }0_{k}\theta_{k}T_{k}\left(\overset{-}{L}\right)c\text{,} \end{array}$$

where c denotes one IoT sensor mounted on a student, $\overset{-}{L}=2/\lambda_{\max}L-I_{d}$, and *λ*max is the maximal eigenvalue of the normalized Laplacian L. The output of the l^th^ layer can then be expressed as

(4) $$\begin{array}{c} o_{s}^{l}=\overset{F_{in}}{\underset{i=1}{\sum }}g_{\theta_{i}^{l}}\left(L\right)c_{s,i}^{l}\text{,} \end{array}$$

where F_out_ and F_in_ denote the output filter and input filter, respectively. *θ*_*i*_^*l*^∈ R_K_ is the Chebyshev coefficient and *x*_*s*,*i*_^*l*^ is the input graph at layer l for student s.

**Figure 2:**
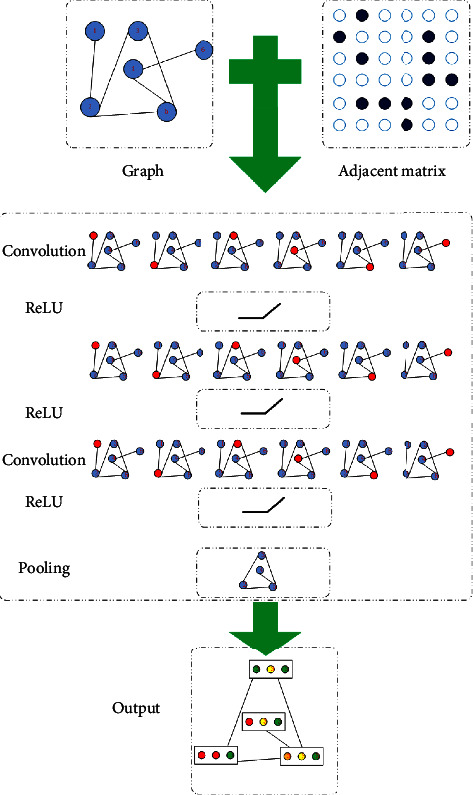
The structure of the proposed GNN.

A pooling layer is located at the end of the proposed GNN. In a fashion of semi-unsupervised learning, the feature map generated from the presented GNN can yield a pairwise association between the subjects. The intact GNN with the pooling operator is demonstrated in Figure 2.

#### 2.2.2. Network Architecture

As described in Table 1, there are 3 pairs of convolutional layers integrated with the rectified linear unit (ReLU) as well as a pooling layer within the proposed GNN architecture. To guarantee the invariant scale for the graphs, the pooling is only incorporated at the end of the convolution operations. The dropout rate of every convolutional layer is 0.5.

In the initial stage, the training rate is set to 0.001 with fixed 500 iterations. Once the decrease in validation accuracy lasts for two consecutive iterations, then the learning rate is multiplied by 0.5. During the training process, 80% of the samples were used as the training set and the remaining 20% were taken as the testing set, and 100 of the training samples were used as the validation set. In total, there were less than 1,000 parameters in the proposed graph network. We trained the whole model by leveraging a back-propagating strategy and no overfitting was observed during training. Furthermore, the expected outcome of the proposed network is the binary status of each student (node in the graph) that is positive or negative.

## 3. Results and Discussion

### 3.1. Performance Evaluation Metrics

In this work, we used accuracy, sensitivity, and specificity in the experiments to measure the performance of the comparing methods.

(1) Sensitivity: the ratio between the number of true positives (TP, the samples are labeled as positive by the teachers; meanwhile, the method generates the correct result) and the number of all of the samples.
  (5) $$\begin{array}{c} Sensitivity=\frac{TP}{TP+FN}\text{,} \end{array}$$
  where FN denotes the false negative (the outcome of one sample labeled as positive is negative).
(2) Specificity: the ratio between the number of true negatives (TN, the outcome of one sample labeled as negative is negative) and the number of all of the samples.
  (6) $$\begin{array}{c} Specificity=\frac{TN}{TN+FP}\text{,} \end{array}$$
  where FP denotes the false positives (the outcome of one sample labeled as negative is positive).
(3) Accuracy: the ratio between the number of correctly identified subjects and the number of all of the samples.
  (7) $$\begin{array}{c} Accuracy=\frac{TP+TN}{TP+FN+TN+FP}\text{.} \end{array}$$

### 3.2. Experimental Results

In this work, the proposed GNN was implemented with the TensorFlow 2.0 deep learning architecture with Python as the programming language.

**Figure 3:**
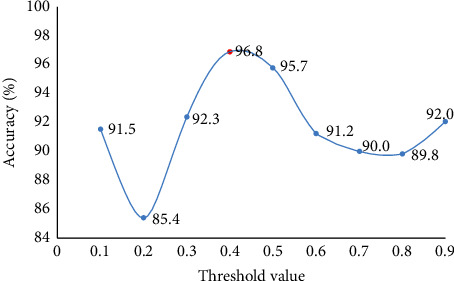
The optimal threshold value set in the proposed GNN.

The edge between each pair of students in the graph would affect the whole structure of the graph. A global threshold is expected to determine if one edge is preserved or not. Thus, we tested the effects of various threshold values to determine the optimal threshold. In the preliminary test, only 9 different numbers were leveraged (0.1, 0.2, ..., 0.9). Meanwhile, to decrease the computation complexity of calculating the optimal threshold value, only the subsamples were used during this stage. According to the outcome from the subsamples in Figure 3, 0.4 is chosen as the threshold value in the following experiments.

Second, we conducted the experiments on the 6,104 samples captured from 16 students by using the proposed approach. For each student, the corresponding number of samples ranged from 197 to 405. It is widely accepted that the quantity and quality of the training samples significantly relate to the performance of machine learning models, as well as the deep learning models.

**Figure 4:**
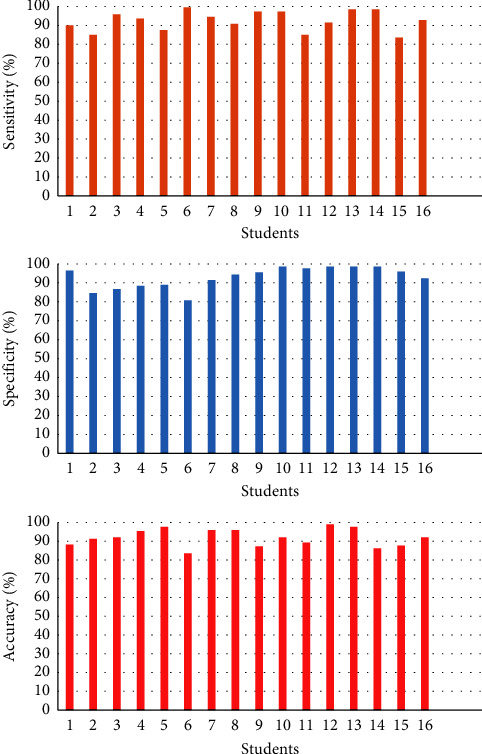
The experimental outcome (sensitivity (%), specificity (%), and accuracy (%)) of the proposed approach for each student.

Learning-based algorithms: accordingly, the sensitivity, specificity, and accuracy yielded from our model can rise or fall for different students in an online class, as shown in Figure 4.

As shown in Figure 4, the accuracy for all of the students is greater than 80% by using the proposed approach, while the accuracy of students 5, 12, and 13 is almost 100%. Moreover, we also examined the samples collected from student 6 whose accuracy is the lowest among the students. We found that student 6 contains the fewest samples (197) compared with the other students.

Generally, the proposed method can provide satisfactory sensitivity and specificity for most of the subjects. However, its performance relates to the quantity and quality of the input samples. By improving the data sample, the proposed GNN can be a potentially valuable instrument for online learning performance evaluation in practice.

**Figure 5:**
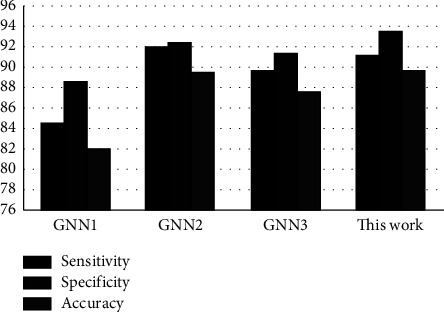
The comparison between the state-of-the-art and our work in terms of sensitivity (%), specificity (%), and accuracy (%).

Third, we conducted the comparison experiments between the state-of-the-art and the proposed framework on the entire dataset that was manually collected. Experimental results as shown in Figure 5 demonstrate that the current work achieved competing performance over the state-of-the-art techniques. Although the sensitivity produced by [37] is better than the proposed approach, this work outperformed the state-of-the-art methods [31, 37, 38] in both the specificity and accuracy of the whole data samples.

## 4. Discussion

The results of the experiments show that the proposed method can provide a favorable outcome for the issue that has an intrinsic graph structure, and it can be useful for other cases rendering similar characteristics. However, there are some limitations of the presented approach that need to be addressed before it is applied to those tasks. For instance, it does not take temporal information into consideration. Therefore, the relations between sequential samples might be neglected by using our approach. Moreover, this method is unable to handle less structured representations.

## 5. Conclusions

In this work, the VR apparatus and IoT sensors were introduced to implement an online music learning platform. By leveraging it, personal information can be collected and formed into a non-Euclidean graph. Since it is difficult to employ the manners commonly adopted in an offline classroom, the identification and prediction of the students' real-time state deserve in-depth research for the online learning scenes. Bearing this in mind, we proposed a spatial-temporal GNN-based framework. Both the interdependent associations between the students and the corresponding development process can be unveiled from the presented GNN model.

To evaluate the performance of the proposed framework, comparison experiments were conducted between the state-of-the-art techniques and the proposed method. Experimental results demonstrated that the combination of VR and IoT, as well as GNN, should be taken as a potentially valuable instrument for online music learning.

In the future, the application of global average pooling (GAP) needs to be studied since it is supposed to decrease the number of parameters and eliminate overfitting and we will continue to delve into the online learning platforms and the applications of various machine learning-based algorithms into them.

## Figures and Tables

**Table 1:** Details of the proposed GNN architecture.

Layer	Conv	ReLU	Conv	ReLU	Conv	ReLU	Pooling	Class
Channels	16	N/A	32	N/A	64	N/A	N/A	2
K-order	9	N/A	9	N/A	9	N/A	N/A	N/A
Stride	1	N/A	1	N/A	1	N/A	N/A	N/A

## Data Availability

The data used to support the findings of this study are available from the corresponding author upon request.
